# Brazil: Two Realities for the Treatment of One Disease

**DOI:** 10.5935/abc.20190093

**Published:** 2019-05

**Authors:** Leonardo Guimarães, Adriano Caixeta

**Affiliations:** 1 Quebec Heart and Lung Institute - Laval University, Quebec - Canada; 2 Escola Paulista de Medicina - Universidade Federal de São Paulo, São Paulo, SP - Brazil; 3 Hospital Israelita Albert Einstein, São Paulo, SP - Brazil

**Keywords:** Coronary Artery Disease, Cardiovascular Diseases/mortality, ST Elevation Myocardial Infarction, Myocardial Revascularization, Diabetes Mellitus, Percutaneous Coronary Intervention, Drug-Eluting Stents

Current drug-eluting stents (DES) are safer and more effective (lower restenosis rates) than bare-metal stents (BMS),^1,2^ and are able to reduce short- and long-term cardiovascular outcomes.^3,4^ In patients with ST segment elevation acute myocardial infarction (STEMI), the randomized study EXAMINATION^5^ evaluated 1,498 patients with STEMI, who were allocated for the treatment of percutaneous coronary intervention (PCI) with new-generation DES containing everolimus or BMS. After a 5-year follow-up, there was a relative reduction of combined outcomes and mortality of 20% and 30%, respectively, in favor of DES. Finally, despite the higher initial cost in the index procedure, DESs present better cost-effectiveness compared to BMS on long-term.^6^ In this context, in 2018, the most current Guideline for coronary artery bypass grafting of the European Society of Cardiology considers recommendation Class I the use of DES in any and all clinical settings, including STEMI.^7^

Cardiovascular disease is the leading cause of death worldwide and diabetes mellitus is one of the most important risk factors for coronary atherosclerotic disease (CAD).^8^ Diabetic patients present 2 to 3-fold higher mortality after acute coronary syndrome compared to non-diabetics.^9^ Besides, as these patients present expressive endothelial dysfunction, high inflammatory response to vascular injury, diffuse CAD and coronary arteries of smaller caliber, they develop higher rates of in-stent restenosis.^10,11^ In view of that, using DES is even more imperative in patients with diabetes, and presents 87% less risk of in-stent restenosis and 77% less risk of revascularization of the target lesion compared to BMS.^12^

In this study VICTIM Oliveira et al.,^13^ analyzing the incorporation of DES in public and private institutions in the State of Sergipe (between 2014 and 2017; after the approval of its use in Brazil's public health system [SUS] at the amount of BRL 2,034.50), found that only 8.7% of diabetic patients with STEMI were treated with DES in the public system, while 90.6% received DES in the private healthcare system. These figures make evident the worrying reality of the Brazilian public health system regarding the treatment of STEMI, especially in a vulnerable population such as diabetics. Moreover, despite the official approval (Ordinance No. 29 issued by the Ministry of Health) of such advanced technology, in Brazil's public healthcare system, its use in non-diabetics and diabetics is important and significantly lower than that of the private healthcare system. In this study, there was no statistical difference for the number of risk factors per patient between the groups, with most of them presenting ≥ 2 cardiovascular risk factors. Noted that the main determinants to receive this globally recommended and proven superior therapy were: family income and education level and, consequently, being able to afford private healthcare. In the United States, in 2003 (one year after DES started being used), 32.7% of diabetic patients undergoing PCI received DES and, in 2011, this number was in excess of 75%.^14^

According to the Brazilian National Health Agency (ANS), in 2019, only 24.3% of Brazilians have private health insurance and, because of that, less than one quarter of the population has access to the treatment recommended by international and Brazilian guidelines.^15^ On the other hand, the vast majority of the population of diabetics only have BMS available. In the international scientific community, unlike the Brazilian reality, the debate about the use of BMS versus DES is now outdated. Also, progress of a new generation of DES such as those of ultrathin struts with bioabsorbable and non-polymeric polymers is discussed.

Thus, as demonstrated in the VICTIM registry, Brazil - a developing country - could be divided into two major nations regarding the treatment of STEMI by PCI: one, the public healthcare system, with most of the population exposed to non-contemporary treatment and unequivocally inferior clinical outcomes; another, the private health system with a population with better socioeconomic conditions and access to the best technologies, similar to those of developed countries. By addressing the deficiencies found in Brazil's public healthcare system in the treatment of a significant portion of the population, this study is expected to stimulate reflections and changes in health promotion and the provision of new treatments to the impoverished population.
